# Prevalence and pattern of antibiotic use and resistance among Iraqi patients: a cross-sectional study

**DOI:** 10.4314/ahs.v24i3.7

**Published:** 2024-09

**Authors:** Hashim Talib Hashim, Ali Talib Hashim, Hossam Tharwat Ali, Haya Mohamed, Ahmed Elrefaey, Ameer Almamoury, Narjiss Aji

**Affiliations:** 1 University of Baghdad, College of Medicine, Baghdad, Iraq; 2 Golestan University for Medical Sciences, Gorgan, Iran; 3 Qena Faculty of Medicine, South Valley University, Qena, Egypt. Qus, Qena 83621, Egypt; 4 Ain Shams University, Faculty of Medicine, Cairo, Egypt; 5 Al Qadisiyah college of Medicine, Al Diwaniyah, Iraq; 6 Faculty of Medicine and Pharmacy of Rabat, Morocco

**Keywords:** Antibiotic, resistance, Iraq

## Abstract

**Background:**

According to The Centers for Disease Control and Prevention (CDC) 2019, around 32,000 deaths in addition 2.8 million infections occur annually in the US because of antibiotic-resistant bacteria.

**Objectives:**

To determine the prevalence and study pattern of antibiotic use and resistance among Iraqi patients.

**Methodology:**

We carried out a cross-sectional study from January 2021 to October 2022 including data of 850 patients at different private and general hospitals, primary health centers, and private clinics. The data was collected during the patient's admission or visiting time using medical records and mini-interviews.

**Results:**

*Escherichia coli* and *Staphylococcus aureus* were the most detected pathogens in our sample in 14.5% and 11.29% of the patients respectively. Most patients (87.18%) had taken over-the-counter antibiotics previously. Around 55% of the antibiotics that were tested were resisted among our patients who were included in 2021. This percentage has increased to about 75% of the included patients in 2022. Amoxicillin was the most resistant antibiotic (77%) in our sample while meropenem was the least resistant among the tested antibiotic (5%).

**Conclusion:**

Antibiotic resistance is a major public health concern that is often caused by the overuse and misuse of antibiotics, as well as poor infection control practices.

## Introduction

Before the discovery of antibiotics many decades ago, infection was by far one of the most pressing threats to the survivability of humans, and the most prominent leading cause of death^1^. The introduction of antibiotics created a dramatic shift in the management of a multitude of infectious diseases that were previously considered incurable^2^. However, over 5,000,000 die each year due to bacterial infections^3^. Yet, only soon later, a crisis stemming from this very breakthrough began to develop as antibiotic resistance became a global public health issue.

This phenomenon has raised the need to develop antibiotic susceptibility tests (ASTs). However, there is still a lack of data regarding the specific application of ASTs results in clinical practice. This has given rise to the “90-60” rule which means that 90% of cases with susceptible organisms will respond clinically well to the tested antibiotics. On the opposite side, 60 % of cases with resistant organisms will show a good response to the tested antibiotics^4^.

According to The Centers for Disease Control and Prevention (CDC) 2019, around 32,000 died in addition, 2.8 million infections occur annually in the US because of antibiotic-resistant bacteria^5^. Higher numbers are reported for patients with many causes of death including bacterial resistance. For example, over 50,000 patients with ventilator-associated pneumonia and 270,000 with sepsis die annually in the US^6^,^7^. Moreover, a systematic analysis from over 200 countries and territories in 2019 revealed that bacterial resistance was responsible for 4.95 million deaths, with around 1.27 deaths directly attributed to bacterial resistance^3^. CDC estimated that in 2025, total deaths from multidrug resistance will exceed 10 million in case there will be no available appropriate antibiotics, which is more than the combined number of deaths from heart diseases and cancer^8^.

Although such a public health issue affects all nations or countries, it is disproportionately higher in low and middle-income countries^9^. Dynamic factors that affect health policies and practices are inseparable from socio-economic, political, cultural, and environmental aspects^10^. Iraq is a low-middle-income country that is located in Asia with around 42.8 million citizens^11^. In the last decades, Iraq has suffered wars and occupation. Thus, Iraqi health systems struggle to keep the momentum after years of conflict^12^. According to the World Health Organization (WHO), There were only 13.2 hospital beds and less than a primary healthcare facility (0.7) per 10,000 population^13^. Moreover, there are no specific regulations on antibiotic dispensing and use among the general population which contributes to the improperly increased misuse of antibiotics^14^,^15^. Moreover, poor knowledge regarding antibiotic misuse among the Iraqi population was also reported in addition to the physicians' and pharmacists' bad attitudes toward the prescription and dispensing of antibiotics^15^,^16^.

Many factors have been attributed to the accelerating rates of antibiotic-resistant bacteria and the most prominent of which is the overuse and misuse of antibiotics in the treatment of bacterial and non-bacterial infections. Failure to comply with prescribed course durations or ultimately resorting to nonprescription antibiotics increases the risk of developing side effects and complications, promotes the evolution of infections difficult to manage, and raises the economic cost of treatment^17^. According to the World Health Organization (WHO), resistance to antibiotics which are considered first-line for empirical therapy for many severe infections e.g. fluoroquinolones and beta-lactam antibiotics constitutes around 70 % of deaths attributable to antibiotic resistance^18^. A thorough investigation into the resistant pathogenic bacterial strains is warranted to curb the evolution of antibiotic resistance and mitigate its consequences. Additionally, understanding the factors fueling antibiotic resistance is a fundamental step in formulating an appropriate approach to slow the propagation of this dilemma.

The main aim of this study is to determine the prevalence and study pattern of antibiotic use and resistance among Iraqi patients. Our study was conducted on patients from a wide range of age groups from pediatric to elderly populations in multiple healthcare settings, with the inclusion of respective data about their antibiotic medication consumption. We analyzed the corresponding patterns of antibiotic resistance in our study subjects between 2021 and 2022.

## Patients and Methods

### Study design, setting, and participants

A cross-sectional study was carried out from January 2021 to October 2022. The study was conducted at different private and general hospitals, primary health centers, and private clinics. The data was collected while the patients were admitted to the hospitals, and during the vising time and diagnosis at primary health centers and private clinics. Any patient, including both males and females from all the regions of Iraq, who was diagnosed with an infection, and did an antibiotic sensitivity test was included. Patients who refused to participate or those with incomplete data were excluded. Data collection was performed based on a convenience sampling type, using the patient's medical record and a mini-interview with the patients. The sample size was calculated using Epi Info statistical calculator 7.2.5. Version, which is a trademark of the Centers for Diseases Control and Prevention (CDC), with the following parameters: a confidence interval of 95%, an expected frequency of 50%, and an acceptable margin of error of 5%. The minimum sample size was 384 responses.

### Study Tool

The questionnaire consisted of two parts. The first, which was done as a mini-interview, included demographic data; age, gender, race, place of residency, work status, monthly income, comorbidities, history related to infections, sources of antibiotics, and comletion of the antibiotic course. The second, which was collected using the patient's medical records in the hospital or the center, consisted of data about microbiological profiles and antibiotic susceptibility.

### Ethical consideration

The ethics approval was secured from the ethical committee at the University of Baghdad, College of Medicine. Every participant agreed to contribute to the study before the mini-interview and the participation was completely voluntary and safe confidentiality was maintained by collecting anonymous responses.

### Statistical analysis

The data collected through the questionnaire was transferred to Excel format and the raw data was encrypted in the Excel sheet to perform a better and more precise statistical analysis on the statistics program. The program used for statistical analysis was SPSS version 25. Categorical baseline variables were denoted as frequencies and percentages, while continuous variables were presented as means and standard deviations (SDs). We used the chisquare test to compare the differences between the study variables and the questionnaires. A p-value of less than 0.05 showed a statistically significant outcome.

## Results

In total, data of 850 patients were included in our analysis. Most of them (66.2%) were males while nearly half of them (46.2%) were above 40 years old. Data of 259 individuals (30.47%) were collected from general hospitals while 230 (27.05%) and 211 (24.82%) were from private clinics and primary health centers respectively. Multiple infections was predominant among the patients (19%) while pneumonia (15.6%) followed by urinary tract infection (11.5%) were the most common single infections. The majority of the patients (87.18%) had taken over-the-counter antibiotics previously while half of them (51%) didn't complete the course of the antibiotics. Around 44% of our patients had more than one antibiotic at a time. The details of the demographic characteristics are summarized in Table 1.

**Table 1:** The demographic data of the included patients

*Variables*	*N. (%)*
***Age***	
≤ 15 years	264 (31.1%)
15 – 40 years	193 (22.7%)
> 40 years	393 (46.2%)
***Gender***	
Male	562 (66.2%)
Female	288 (33.8%)
**Place of admission or visit**	
Private hospital	150 (17.66%)
Public health center	211 (24.82%)
General hospital	259 (30.47%)
Clinic	230 (27.05%)
***Duration of admission***	
≤ 14 days	521 (61.3%)
> 14 days	329 (38.7%)
***Type of infection***	
Pneumonia	132 (15.5%)
Urinary tract infection (UTI)	98 (11.6%)
Gastroenteritis	127 (15%)
Sore throat	47 (5.53%)
Meningitis	71 (8.35%)
Bone infection	36 (4.23%)
Skin infection	46 (5.4%)
Others	128 (15.05%)
Multiple infections	165 (19.34%)
***Did you have fever?***	
Yes	741 (87.2%)
No	109 (12.8%)
***Co-morbid conditions:***	
Diabetes mellites (DM)	321 (37.85%)
Hypertension (HTN)	111 (13.05%)
Heart diseases	47 (5.53%)
Cancer	12 (1.41%)
Autoimmune diseases	17 (2%)
Genetic diseases	27 (3.17%)
Others	11 (1.3%)
None	304 (35.76%)
***Place or residency:***	
Rural	470 (55.3%)
Urban	380 (44.7%)
***Race***	
Arabic	615 (72.35%)
Kurdish	147 (17.3%)
Turkmen	88 (10.35%)
***Did you get the infection in the hospital?***	
Community-acquired	413 (48.6%)
Nosocomial infection	437 (51.4%)
***Work***	
Full-time job	325 (38.25%)
Part-time job	165 (19.4%)
Unemployed	360 (42.35%)
***Monthly income***	
≤ 250K Iraqi Dinar (IQD)	478 (56.23%)
250K – 500K IQD	102 (12%)
500k – 1 million IQD	198 (23.3%)
> 1 million IQD	72 (8.47%)
***Number of admissions during the last six month:***	
Only the present one	632 (74.4%)
***Number of admissions during the last six month:***	
Only the present one	632 (74.4%)
More than 1 admission	218 (25.6%)
***Have you taken over-the-counter prescription of antibiotic?***	
Yes	741 (87.18%)
No	109 (12.82%)
***Who prescribed you the antibiotic previously?***	
Doctor	209 (24.6%)
Pharmacist	125 (14.7%)
Nurse	77 (9.05%)
Yourself	105 (12.35%)
Family member	334 (39.3%)
***Have you completed the course of the antibiotic?***	
Yes	416 (48.95%)
No	434 (51.05%)
***Have you taken more than one antibiotic at the same time?***	
Yes	378 (44.5%)
No	472 (55.5%)
***Have you suffered from any side effects related to the medications?***	
Yes	364 (42.83%)
No	486 (57.17%)

Based on the culture and sensitivity results, *Escherichia coli*, followed by *Staphylococcus aureus* and *Enterococcus* species were the most detected pathogens in our sample in 14.5%, 11.29%, and 8.7% of the patients respectively. Amoxicillin was the most resistant antibiotic (77%) in our sample while meropenem was the least resistant among the tested antibiotic (5%). Erythromycin and Trimethoprim-sulfamethoxazole were resisted among 63.8% of the samples. Tetracycline and ampicillin were resisted among 63.5% and 50.8% of the patients respectively. Based on the culture and sensitivity analyses, around 80% of the included patients were prescribed multiple anibiotics. The details of the microbiological assessment of the patients are summarized in Table 2.

**Table 2:** The data related to the culture and resistance investigations

*Variables*	*N. (%)*
***The type of microbe detected:***	
*Escherichia coli*	123 (14.5%)
*Staphylococcus aureus*	96 (11.29%)
*Enterococcus species*	74 (8.7%)
*Pseudomonas aeruginosa*	15 (1.76%)
*Candida Albicans*	13 (1.53%)
*Staphylococcus epidermidise*	19 (2.23%)
*Streptococcus Pneumonia*	32 (3.7%)
*Streptococcus pyogenes*	21 (2.47%)
*Staphylococcus Saprophyticus*	11 (1.29%)
*Klebsiella Pneumonia*	11 (1.29%)
*Streptococcus faecalis*	20 (2.35%)
*Hemophilus influenza*	23 (2.7%)
*Salmonella typhi*	32 (3.7%)
*Clostridium species*	10 (1.17%)
Mycobacterium species	10 (1.17%)
Campylobacter Species	9 (1.05%)
Shigella species	55 (6.4%)
Others	276 (32.4%)
***Type of antibiotic resistance:***	
Meropenem	42 (5%)
Gentamycin	103 (12.11%)
Ciprofloxacin	345 (40.58%)
Amikacin	365 (43%)
Imipenem	120 (14.11%)
Cefotaxime	209 (24.58%)
Cefepime	102 (12%)
Tetracycline	540 (63.5%)
Ampicillin	432 (50.8%)
Piperacillin	230 (27.05%)
Ceftriaxone	235 (27.64%)
Ceftazidime	120 (14.11%)
Vancomycin	345 (40.5%)
Oxacillin	235 (27.64%)
Amoxicillin	654 (77%)
Erythromycin	543 (63.8%)
Levofloxacin	347 (40.8%)
Clarithromycin	234 (27.5%)
Trimethoprim-sulfamethoxazole	543 (63.8%)
***Multiple antibiotic prescription?***	
Yes	687 (80.83%)
No	163 (19.17%)
***Discharge after treatment:***	
≤ 7 days	432 (50.8%)
> 7 days	418 (49.2%)

**Figure 1:**
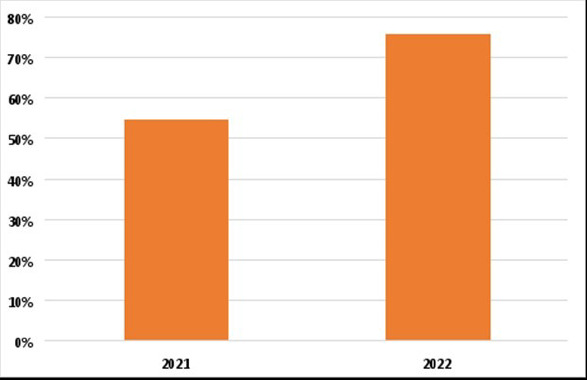
Percentage of resisted antibiotics in 2021 vs 2022

We found that about 55% of the antibiotic that was tested were resisted among our patients who were included in 2021. This percentage has increased to about 75% of the included patients in 2022 (Figure 1).

We found statistically significant differences between the age group in the type of detected pathogens in the culture with a p-value of 0.002. Among the < 15 years old group, *E. coli, Shigella*, and *H. influenza* were the most common to be detected in culture. On the other hand, *staphylococcus aureus, streptococcus pneumoniae*, and *salmonella typhi* were the most detected in the 15-40 years old group. Patients older than 40 years had *enterococcus species* and *streptococcus pyogenes* as the most detected. There were also statistically significant differences between the age group in the place of admission or visit as follows; public health centers, general hospitals, and clinics were common among the < 15 years group, clinics, and general hospitals for both 15-40 and > 40 years old groups (p-value=0.03).

The general hospitals and clinics had a higher probability for the patients to complete the course, unlike the public health center with a higher probability of not completing the course (p-value = 0.001). Moreover, general hospitals and clinics as the most to prescribe antibiotics, while public health centers are the least (p-value = 0.04). Similarly, there were statistically significant differences between the place of admission or visit and the side effects suffered from (p-value = 0.03) with the general hospitals and clinics having a higher number of side effects suffered from.

On the other hand, there were no statistically significant differences between the type of microbe detected and the race (p-value = 0.8), monthly income (p-value = 0.06), place of residence (p-value = 0.07), and gender (p-value = 0.5).

## Discussion

To our knowledge, this is the first comprehensive study to entail a descriptive report of resistant patterns expressed by bacterial pathogens affecting different age groups in Iraq. With a large sample size of 850 participants collected within less than two years, this multicentric study lays the foundation for upcoming research to routinely assess microbiological profiles of resistant strains and recognize emerging patterns of antibiotic susceptibility in the nation. Our results concluded that over 55 % and 75 % of antibiotics tested were resisted in our patients during the years 2021 and 2022 respectively. Thus, surveillance and monitoring efforts are pivotal to diminishing the upward trend in developing countries with scarce resources, like Iraq, allowing legislators and medical professionals to tailor interventional strategies.

### Microbiological Profile

An investigation into the microbiological profiles of isolated bacteria in our study was performed. The most frequently isolated bacteria were Escherichia coli, followed by Staphylococcus aureus. This is consistent with the findings of a study in the Dominican Republic in 2020 and another in Yemen in 2019, both showing *Escherichia coli* and *Staphylococcus aureus*, as the two most encountered based on samples of 1552 and 412 participants, respectively^19^,^20^. The Yemeni study showed that *staphylococcus aureus* and *E. Coli* were detected in 172 patients (41.74%) and 164 patients (39.8%) respectively^11^. Similarly, *E. Coli* and *staphylococcus aureus* were detected in 363 (23.4%) and 173 (11.1%) samples study conducted in the Dominican Republic^12^. Similar observations were documented in an Ethiopian cross-sectional study, using waste and wastewater samples collected primarily from health institutions^21^.

The predominance of *Escherichia coli* and *Staphylococcus aureus* infections in Iraq, the Dominican Republic, Yemen, and Ethiopia can be interpreted by overlooked reasons beyond the inappropriate use of antibiotics and lack of surveillance systems. In low and middle-income countries, overcrowded and suboptimal sanitary conditions are likely to contribute to the propagation of resistant strains of commensal *Escherichia coli*^22^. Likewise, inadequate application of infection control protocols in healthcare facilities creates a surge in *Staphylococcus aureus* infections, particularly methicillin-resistant strains^23^. Another important nosocomial-acquired infection is Enterococcus, which was the third frequently isolated pathogen in our study. Resistance is particularly common in hospitalized patients in the intensive care unit, emphasizing the urgency for the rational use of antibiotics^24^.

### Antibiotic susceptibility

Patterns of antibiotic resistance in our study were also recorded. Amoxicillin showed an alarmingly elevated resistance of 77%, followed by co-trimoxazole (trimethoprim-sulfamethoxazole), tetracycline, and erythromycin with a figure of 63.8% in all three. On the other hand, studies in Yemen showed that the highest resistance was to co-trimoxazole with antibiotic nonsusceptibility rates reaching 117 resistant cases out of 160 samples (73.12%), surpassing amoxicillin (with clavulanate) which came second with 118 resistant out of 181 samples (65.19%)^19^. Yet, both medications still exhibited the highest overall resistance in Yemen and Iraq, which can be attributed to their widespread use as empirical therapies. An Ethiopian study of medical waste and astewater also showed high resistance to co-trimoxazole (63.1%), but contrary to our results in Iraq and the study in Yemen, tetracycline recorded the highest resistance in Ethiopia, followed by co-trimoxazole and ampicillin (62.3%)^21^. A recent study in Libya on *E. Coli* isolates from outpatient urine samples showed that ceftriaxone and co-trimoxazole had the highest resistance rates of 62.5% and 54.8% respectively. Around 47% and 15.4% of the samples were resistant to amoxicillin and levofloxacin respectively in the same study^25^. In our study, moderate resistance was seen to ampicillin (50.8%) and levofloxacin (40.8%). However, levofloxacin demonstrated the lowest resistance rates in Yemen, with 21 resistant samples out of 135 (15%)^19^.

### Source of antibiotics

The feasibility of obtaining antibiotics is a growing problem worldwide, particularly in developing countries like Iraq, where the practice is hugely unregulated. Patients can purchase and dispense antibiotic drugs without prescriptions, bypassing any authorized healthcare service. The WHO has long advocated for the restrictive use of antibiotics, for symptoms consistent with a bacterial infection, at the discretion of the treating clinician. However, our study revealed that over 85% (741) of participants acquired over-the-counter antibiotics and 75% (641) did not have a doctor's prescription. In contrast with neighboring countries in the Middle East, data on the Iraqi population is markedly unfavorable, with studies in Kuwait reporting the use of non-prescribed antibiotics to be just over 25%^26^, and Oman below 20%^27^. Eastern Europe recorded the highest numbers in the region, with self-medication rates reaching their peak at 19.8% and 21% in Romania and Slovenia respectively^28^, but those numbers are also a fraction of Iraq's figures. Furthermore, a study in the United States reported a rate of as little as 5% in an ethnically and socioeconomically diverse population^29^. Usage of non-prescribed antibiotics is especially higher in the Latino community, which imports them across the border or obtains them locally but discreetly^30^.

Our study found that 39% (334) of participants initiated antibiotic treatment solely on the advice of family members, contravening public health recommendations. Family and friends were thus the most common source of antibiotics in our study, surpassing doctors (24.6%), pharmacists (14.7%), and nurses (9.05%). The number of patients who obtained antibiotics from family and friends is substantially less in countries like the United Kingdom (2.9%)^31^. Similarly, a study conducted in Kuwait and another in 19 European countries each showed only 8% antibiotic acquisition from family and friends^26^,^28^. Accordingly, data on antibiotic misuse and overuse in the Iraqi population demands tailored intensive strategies to address social, economic, and regulatory barriers to doctor consultation on antibiotic prescriptions.

### Completion of the course

Another aggravating factor to antibiotic resistance is an incomplete antibiotic course. Patients who do seek medical attention do not always adhere to treatment instructions. In our study, over half of study subjects (51.05%) failed to take their full prescribed course. Nonetheless, studies performed in Middle Eastern countries indicated fewer rates of non-compliance; only 29% of Kuwaiti respondents were unkeen on completing the course and 37% stopped when their symptoms improved^26^. In another study in Oman, 31% discontinued treatment earlier than intended and 32.5% missed doses^27^. Kuwait and Oman's results are in accordance with a study in Hong Kong, China which showed a 30% rate of non-adherence to the course timeline^32^. Therefore, compliance with antibiotic prescriptions in Iraq needs to be improved, by filing patients' knowledge gaps and incorporating engaging aids across the continuum of care, to deflate the plight of antibiotic resistance.

Yet, non-compliance with prescribed regimens is a complex phenomenon, with multiple influences intertwining together. Patients often alter the dosing intervals, with some inadvertently or negligently omitting single doses, and others deliberately adding under the assumption that recovery will be quicker with higher doses. Patients were also found to prematurely stop their treatment following the resolution of symptoms, as seen in studies in Kuwait and Oman. Others attribute early termination of antibiotic treatment to uncomfortable or painful side effects, as seen in a study carried out in China (15.6%)^32^.

### Impact of Place of Admission or visit on Compliance, Number of Antibiotics, Side Effects

Aside from the aforementioned factors, further intricacies that heighten antibiotic resistance are yet to be explored. In our study, for example, the patient's place of admission or visit was found to have a profound impact on course completion (p-value = 0.001), the number of antibiotics administered (p-value = 0.04), and side effects experienced (p-value = 0.03), with statistically significant results in all three. To illustrate, patients who were admitted to general hospitals and who visited clinics in Iraq were more likely to finish the course as recommended but were prescribed more antibiotics and experienced more side effects, as opposed to those in primary health centers. Even with patient compliance to course completion, concomitant administration of multiple drugs can have grim implications on the transmission of antibiotic-resistant strains. It is also worth mentioning that 44.5% (378) were given multiple antibiotics and 42.38% (364) reported side effects, which brings into question the potential relationship between the clinical use of antibiotics in combination and the development of adverse effects.

## Strengths and limitations of the study

Our study was done in many different patient settings with different health conditions, age, and economic status. This has allowed us to conclude some differences across subgroups in each variable. Thus, more targeted procedures regarding each subgroup would be effective against this serious phenomenon. Despite that, some limitations did exist. This study did not extend its scope to encompass the impact on human health due to antibiotic utilization in livestock and agricultural products which can potentially cause more health and economic damage. The convenience sampling method also limits the generalizability of the results. Comparisons with other countries in different categories were limited to the ones with similar study objectives and sample size. Moreover, some information was gathered using direct questions to the patients, which may introduce recall bias, as most surveys do.

## Conclusion

Antibiotic resistance is a serious problem that can have significant negative impacts on public health. Many commonly used antibiotics such as amoxicillin and erythromycin are having a rising incidence of resistance among Iraqi patients. Inappropriate practices of the population are significantly contributing to this serious issue. These practices include unnecessary use of antibiotics without a physician's recommendation and improper duration of use. Thus, raising public awareness about the situation and its implication on their health is urgently needed. It is important to take steps to reduce the spread of antibiotic resistance and to preserve the effectiveness of existing antibiotics.

### Recommendations and implications for clinical practice and future research

There is a growing need for an international, collaborative effort that prioritizes the surveillance and monitoring of antibiotic consumption. Thorough studies exploring the prevalence of antibiotic resistance, particularly in developing countries like Iraq, and the overlooked factors contributing to the rising phenomenon can enable an accurate assessment of the efficacy of current strategies and lay out the framework for future interventions advocating for rational antibiotic use. Further research is essential to continuously evaluate patterns of antibiotic susceptibility and microbiological epidemiology of isolated profiles in different medical settings in Iraq. Additionally, this paper calls for the investigation of susceptibility factors to certain isolates in relevance to age and place of admission, which will push forward strategies to combat the overuse and misuse of antibiotics in specific populations. Lastly, assessing and monitoring patients admitted into or visiting different medical facilities is imperative. Such endeavors hold paramount promising benefits for the current generation and the ones to come.

## Data Availability

Data generated and analyzed during this study are available upon request.

## List of Abbreviations

ASTs: Antibiotic susceptibility tests
CDC: Centre for Disease Control and Prevention
US: the United States
WHO: the World Health Organization
